# The Obsteteric Memoirs and Contributions of James G. Simpson, M. D., F. R. S., Prof. of Midwifery in the University of Edinburgh

**Published:** 1856-01

**Authors:** 


*The Obsteteric Memoirs and Contributions of James G. Simpson, M.D., F.R.S., Prof. of Midwifery in the University of Edinburgh.* Edited by W. D. PRIESTLY, M. D., Edinburgh, formerly Vice President of the Parisian Medical Society; and HORATIO R. STORER, M. D., Boston, U. S., one of the Physicians to the Boston Lying-in Hospital, &c. Vol. I. Philadelphia: J. B. Lippincott & Co., 1855.

It is unnecessary for us to say one word in commendation of this collection of Dr. Simpson's Essays. His name is indisolubly connected with the department of medicine to which he has devoted his life. His admirers in this country—and their name is legion—will be glad to have an opportunity of becoming still better acquainted with him, through this volume.

Dr. Storer, the American Editor, has long been intimately acquainted with the author, and is, perhaps, the most suitable person that could have been selected, on this side of the Atlantic, for the execution of the task assigned him.

For sale by S. C. Griggs & Co. J.

